# Behavioural intervention for adolescent uptake of family planning: a randomized controlled trial, Uganda

**DOI:** 10.2471/BLT.20.285339

**Published:** 2021-09-29

**Authors:** Sara Flanagan, Arielle Gorstein, Martha Nicholson, Stephanie Bradish, Diana Amanyire, Andrew Gidudu, Francis Aucur, Julius Twesigye, Faith Kyateka, Samuel Balamaga, Alison Buttenheim, Emily Zimmerman

**Affiliations:** a ideas42, 80 Broad St 30Fl, New York, NY 10004, United States of America (USA).; b MSI Reproductive Choices, London, England.; c Marie Stopes Uganda, Kampala, Uganda.; d University of Pennsylvania School of Nursing, Philadelphia, USA.

## Abstract

**Objective:**

To evaluate the impact of a peer-referral and clinic welcome programme for reducing barriers to adolescents’ uptake of family planning services in Uganda.

**Methods:**

We developed an intervention using behavioural design and carried out a stratified, randomized controlled evaluation of the intervention in girls aged 15–19 years. Sexual and reproductive health clinics were randomized into control (56 clinics) and intervention groups (60 clinics). All intervention clinics received the core intervention (materials to create an adolescent-friendly environment and referral cards to give to friends), while a subset of clinics additionally received training in youth-friendly service provision. We collected clinics’ routine data on monthly numbers of visits by adults and adolescents over a 15-month baseline and 6-month intervention period, 2018–2020.

**Findings:**

In multivariate regression analysis we found significant effects of the intervention on primary outcomes in the pooled intervention group compared with control. Mean monthly visits by adolescents increased by 45% (incidence rate ratio, IRR: 1.45; 95% confidence interval, CI: 1.14–1.85), or over five additional adolescent clients per clinic per month. The mean adolescent proportion of total clients improved by 5.3 percentage points (95% CI: 0.02–0.09). Within treatment arms, clinics receiving the training in youth-friendly service provision showed the strongest effects: a 62% increase (IRR: 1.62; 95% CI: 1.21–2.17) in adolescent clients, or over seven additional adolescents per clinic per month, relative to the control group.

**Conclusion:**

A behavioural change intervention designed to target identified barriers can increase adolescents’ uptake of family planning counselling and services.

## Résumé

**Objectif:**

Évaluer dans quelle mesure un programme d'accueil clinique basé sur l'orientation confraternelle peut contribuer à surmonter les obstacles à l'adoption des services de planification familiale chez les adolescents en Ouganda.

**Méthodes:**

Nous avons mis au point une intervention en nous aidant du design comportemental, puis avons mené une évaluation randomisée contrôlée et stratifiée de cette intervention chez les jeunes filles âgées de 15 à 19 ans. Plusieurs cliniques de santé sexuelle et reproductive ont été réparties aléatoirement dans un groupe de contrôle (56 cliniques) ou d'intervention (60 cliniques). Toutes les cliniques appartenant au second groupe ont reçu l'intervention de base (des ressources servant à créer un environnement adapté aux adolescents, ainsi que des cartes de référence à distribuer autour d'eux), tandis qu'un sous-groupe de cliniques a suivi une formation spécialisée dans la prise en charge des jeunes. Nous avons récolté les données courantes des cliniques relatives au nombre mensuel de visites effectuées par des adultes et adolescents sur une période initiale de 15 mois et une période d'intervention de 6 mois, entre 2018 et 2020.

**Résultats:**

L'analyse de régression multivariée nous a permis de constater que le programme avait d'importantes répercussions sur les résultats primaires au sein du groupe d'intervention par rapport au groupe de contrôle. Le nombre mensuel moyen de visites effectuées par des adolescents a augmenté de 45% (rapport de taux d'incidence, RTI: 1,45; intervalle de confiance de 95%, IC: 1,14–1,85), l'équivalent d'au moins cinq patients adolescents supplémentaires par clinique chaque mois. Le pourcentage moyen d'adolescents sur l'ensemble des patients a connu une hausse de 5,3 points de pourcentage (IC de 95%: 0,02–0,09). Dans le groupe d'intervention, les cliniques ayant suivi une formation spécialisée dans la prise en charge des jeunes ont enregistré une progression spectaculaire de 62% (RTI: 1,62; IC de 95%: 1,21–2,17) auprès des patients adolescents, ce qui signifie que chaque clinique a accueilli au moins sept adolescents supplémentaires par mois, par rapport au groupe de contrôle.

**Conclusion:**

Une intervention induisant un changement de comportement, conçue dans le but de déjouer des obstacles clairement identifiés, peut favoriser l'adoption des services et consultations de planification familiale chez les adolescents.

## Resumen

**Objetivo:**

Evaluar el impacto de un programa de acogida en clínicas y de referencia entre pares para reducir las barreras que impiden adoptar los servicios de planificación familiar entre los adolescentes en Uganda.

**Métodos:**

Se desarrolló una intervención mediante un diseño conductual y se llevó a cabo una evaluación aleatorizada, controlada y estratificada de la intervención en mujeres de 15 a 19 años. Las clínicas de salud sexual y reproductiva se dividieron de manera aleatoria en grupos de referencia (56 clínicas) y de intervención (60 clínicas). Todas las clínicas de intervención recibieron la intervención principal (materiales para crear un entorno amigable con los adolescentes y tarjetas de referencia para entregar a los amigos), mientras que un subconjunto de clínicas recibió además formación en la prestación de servicios adaptados a las necesidades de los jóvenes. Se recopilaron los datos rutinarios de las clínicas sobre el número de visitas mensuales de adultos y adolescentes durante un periodo basal de 15 meses y un periodo de intervención de 6 meses entre 2018 y 2020.

**Resultados:**

En el análisis de regresión multivariante se encontraron efectos significativos de la intervención sobre los resultados primarios en el grupo de intervención agrupado en comparación con el de referencia. La media de visitas mensuales de los adolescentes aumentó en un 45 % (razón de tasas de incidencia, IRR: 1,45; intervalo de confianza del 95 %: IC: 1,14-1,85), es decir, más de cinco clientes adolescentes adicionales por clínica al mes. El porcentaje medio de adolescentes sobre el total de clientes mejoró en 5,3 puntos porcentuales (IC del 95 %: 0,02-0,09). Dentro de los grupos de tratamiento, las clínicas que recibieron la formación en la prestación de servicios adaptados a las necesidades de los jóvenes mostraron los efectos más fuertes: un aumento del 62 % (IRR: 1,62; IC del 95 %: 1,21-2,17) en los clientes adolescentes, es decir, más de siete adolescentes adicionales por clínica al mes, en relación con el grupo de referencia.

**Conclusión:**

Una intervención orientada al cambio de conducta, diseñada para abordar las barreras identificadas, puede hacer que los adolescentes acepten el asesoramiento y los servicios de planificación familiar.

## ملخص

الغرض تقييم تأثير إحالة الأقران، وبرنامج الترحيب بالعيادة، لتقليل الحواجز التي تحول دون استيعاب المراهقين لخدمات تنظيم الأسرة في أوغندا.

الطريقة قمنا بتطوير تدخل باستخدام التصميم السلوكي، وتنفيذ تقييم تدريجي عشوائي خاضع للتحكم للتدخل لدى الفتيات اللاتي تتراوح أعمارهن بين 15 و19 عامًا. تم ترتيب عيادات الصحة الجنسية والإنجابية بشكل عشوائي إلى مجموعة خاضعة للتحكم (56 عيادة)، ومجموعات للتدخل (60 عيادة). جميع عيادات التدخل تلقت التدخل الأساسي (مواد لإنشاء بيئة صديقة للمراهقين وبطاقات إحالة لمنحها للأصدقاء)، بينما تلقت مجموعة فرعية من العيادات بالإضافة إلى ذلك تدريباً على تقديم خدمة صديقة للشباب. قمنا بجمع البيانات الروتينية للعيادات حول الأعداد الشهرية للزيارات التي يقوم بها البالغون والمراهقون على مدى 15 شهرًا، وفترة تدخل مدتها 6 أشهر، خلال الفترة من 2018 إلى 2020.

النتائج في تحليل للتحوف متعدد المتغيرات، وجدنا تأثيرات ملموسة للتدخل على النتائج الأولية في مجموعة التدخل المجمعة، مقارنةً بمجموعة التحكم. زاد متوسط الزيارات الشهرية بواسطة المراهقين بنسبة 45% (نسبة معدل وقوع الحالة (IRR): 1.45؛ وبفاصل ثقة مقداره 95%: 1.14 إلى 1.85)، أو أكثر من خمسة عملاء مراهقين إضافيين لكل عيادة شهريًا. تحسن متوسط نسبة المراهقين من إجمالي العملاء بمقدار 5.3 نقطة مئوية (وبفاصل ثقة مقداره 95%: 0.02 إلى 0.09). ضمن أذرع العلاج، أظهرت العيادات التي تتلقى التدريب في تقديم الخدمات الصديقة للشباب أقوى التأثيرات: زيادة بنسبة 62% (نسبة معدل وقوع الحالة IRR): 1.62)؛ ‏بفاصل ثقة 95%: 1.21 إلى 2.17) في العملاء المراهقين، أو أكثر من سبعة مراهقين إضافيين لكل عيادة شهريًا، بالنسبة لمجموعة التحكم.

الاستنتاج يمكن أن يؤدي تدخل التغيير السلوكي، المصمم لاستهداف العوائق المحددة، إلى زيادة استيعاب المراهقين لاستشارات وخدمات تنظيم الأسرة.

## 摘要

**目的:**

旨在评估为乌干达青少年接受计划生育服务减少障碍而施行的同行转诊和“诊所欢迎计划”的影响。

**方法:**

我们使用行为设计开发了一项干预措施，并对 15-19 岁女孩的干预措施进行分层、随机对照评估。将性健康与生殖健康诊所随机分为对照组（56 个诊所）和干预组（60 个诊所）。所有干预组诊所都接受了核心干预（创建青少年友好环境的资料和可赠送朋友的转诊卡），同时一部分诊所还接受了青少年友好服务的培训。我们收集了 2018 至 2020 年 15 个月基线和 6 个月干预期间成人和青少年每月就诊次数的诊所常规数据。

**结果:**

在多元回归分析中，我们发现与对照组相比，汇总后干预组的干预对主要结果有显著影响。青少年每月平均就诊次数增加了 45%（发生率比值，IRR：1.45；95% 置信区间：CI: 1.14–1.85)，或每月每个诊所增加超过五个青少年客户。总客户中青少年的平均比例提高了 5.3 个百分比（95% CI: 0.02–0.09）。在治疗组中，接受青少年友好服务培训的诊所显示出最显著的效果：青少年客户增加 62% (IRR: 1.62; 95% CI: 1.21–2.17)，或相比于对照组，每月每个诊所增加超过七个青少年。

**结论:**

针对已识别的障碍实施行为变化干预可以增加青少年接受计划生育咨询和服务的程度。

## Резюме

**Цель:**

Оценить влияние программы по обращению к специалистам по принципу «сверстник-сверстнику» и ознакомлению с клиникой для уменьшения препятствий, мешающих подросткам пользоваться услугами по планированию семьи в Уганде.

**Методы:**

Авторы разработали мероприятие с использованием поведенческого дизайна и провели стратифицированную рандомизированную контролируемую оценку данного мероприятия среди девушек в возрасте 15–19 лет. Клиники сексуального и репродуктивного здоровья были рандомизированы на контрольные (56 клиник) и экспериментальные (60 клиник). Все экспериментальные клиники получили основные средства (материалы для создания благоприятной для подростков среды и направления к специалисту для распространения среди друзей), а отдельная подгруппа клиник дополнительно прошла обучение по оказанию услуг, учитывающих потребности молодежи. Мы собрали стандартные данные клиник о ежемесячном количестве посещений взрослых и подростков в течение 15-месячного исходного и 6-месячного экспериментального периода, 2018–2020 гг.

**Результаты:**

Во время многомерного регрессионного анализа мы обнаружили значительное влияние мероприятия на первичные исходы в объединенной экспериментальной группе по сравнению с контрольной группой. Среднее ежемесячное посещение подростками увеличилось на 45% (отношение частоты случаев, ОЧС: 1,45; 95%-й доверительный интервал: ДИ: 1,14–1,85) или более чем на пять дополнительных клиентов-подростков на клинику в месяц. Средняя доля подростков в общем числе клиентов увеличилась на 5,3 процентных пункта (95% ДИ: 0,02–0,09). Среди групп воздействия клиники, прошедшие обучение по оказанию услуг, учитывающих потребности молодежи, показали самое сильное воздействие: рост на 62% (ОЧС: 1,62; 95% ДИ: 1,21–2,17) среди клиентов-подростков, или более семи дополнительных подростков на клинику в месяц по сравнению с контрольной группой.

**Вывод:**

Мероприятие по изменению поведения, направленное на устранение выявленных препятствий, может повысить интерес подростков к консультированию и услугам по планированию семьи.

## Introduction

Unplanned pregnancy can have a major impact on an adolescent’s health and economic future. Each year, adolescents in low- and middle-income countries have an estimated 21 million pregnancies and 12 million of them give birth;^1^ pregnancy and childbirth complications are the leading cause of death for girls aged 15–19 years.^2^ Surveys in sub-Saharan Africa find that nearly all adolescent girls who have ever been pregnant are not attending school, with pregnancy cited as the most common reason for dropout.^3^ Despite these risks, use of modern family planning methods among adolescents remains low in many places.^4^

In the 2016 Demographic and Health Survey, one in four adolescent girls aged 15–19 years in Uganda had begun childbearing, yet nearly half of births to this age group were reported as mistimed or unwanted, a higher proportion than in older women.^5^ Women who gave birth more than once as an adolescent increasingly report preferring they had their second child at a later age.^6^ Adolescents also have the highest abortion rate among recently sexually active women in Uganda,^7^ exposing them to risks associated with unsafe methods of abortion. Only 39% of sexually active girls aged 15–19 years (253 000 out of 648 000) who do not want a child for at least 2 years use modern contraception, leaving six in 10 girls with an unmet need for contraception.^8^

Increased efforts are needed to ensure not only that contraception is available but that adolescents can access youth-friendly counselling and services to support their informed choices. Interventions aimed at adolescents still forming their identities and understanding of norms around sexuality and gender may be especially important in contexts where social stigma can be a barrier to contraception uptake. Among this age group, peers can be especially influential on relationships and sexual behaviours,^9^ and may be an important barrier or facilitator for access to family planning. However, common interventions such as peer education and youth centres have not been effective at improving adolescents’ access to services and changing their behaviour.^10^

Behavioural design is a systematic approach to intervention development^11^ and a promising strategy to improve sexual and reproductive health-seeking behaviour and outcomes.^12^ The approach involves first diagnosing barriers preventing uptake of a behaviour followed by development of tailored solutions. Employing this approach, we designed an intervention to increase adolescent girls’ uptake of family planning counselling and services in Uganda. The intervention aimed to address behavioural barriers (Table 1) identified through clinic-based observations and qualitative interviews with married and unmarried adolescents, service providers, community health mobilizers and local nongovernmental organization staff. We refined the intervention through collaboration and testing with users, resulting in a multicomponent health behaviour intervention. For this study we evaluated the impact of the intervention on the number of adolescent clients accepting family planning services and the proportion of total clinic clients who were adolescents.

**Table 1:** Behavioural barriers and design objectives for an intervention to reduce barriers to adolescents’ uptake of family planning services, Uganda

Insight	Behavioural barriers	Design objectives
By default, adolescents are not prompted to decide about family planning	• Using family planning implies actively preparing for sex, which is at odds with what adolescent girls perceive as appropriate for them • Adolescent girls do not perceive themselves as having full responsibility or autonomy when it comes to family planning and instead defer to their male partners	• Create an opportunity for adolescent girls to consider whether to use family planning
Social stigma surrounding family planning leads adolescents to overestimate the unpleasantness and visibility of the uptake process	• Adolescent girls perceive greater social stigma from using family planning than from being sexually active but not using family planning • Adolescent girls may overestimate the extent to which their actions to take up family planning are visible to those around them • Adolescents anticipate the uptake process will be unpleasant	• Help girls to envision using family planning as consistent with a positive self-image • Reinforce providers’ commitment to welcome girls and treat them well • Create discreet signals allowing girls to see that others like them consider or use family planning
Adolescents worry about the perceived risks of using family planning	• Adolescents are exposed to inaccurate information that suggests family planning is risky for their health and fertility • Health providers are not always trusted by adolescents, and the peers and community members that adolescents do trust do not always provide accurate information.	• Communicate that girls are welcome at clinics • Create a pathway for girls to learn more about family planning by visiting a clinic, without feeling immediate pressure to take up a contraceptive method
Whether an adolescent perceives tangible losses from unintended pregnancy shapes receptivity to family planning	• Although adolescents may want to avoid pregnancy, they often perceive the risk of pregnancy as more distant and uncertain than they perceive the risks of using family planning • Adolescents who perceive a specific, tangible loss associated with an unintended pregnancy may be more receptive to family planning	• Encourage communication between peers about the reasons they may use or consider family planning
Adolescents do not consider all family planning methods that might be relevant to their needs	• Family planning is commonly viewed as appropriate only for older or married women, while condoms (which are harder to use consistently and effectively) are often considered by adolescents to be the only relevant option for them	• Encourage girls to receive counselling so they can learn about all contraceptive methods that might meet their needs
Adolescents who intend to use family planning sometimes do not follow through	• Some adolescents intend to take up family planning, but do not act on their intention • Some adolescents change their mind or are deterred by worries that the process will be unpleasant or will have social consequences	• Build (or strengthen) an intention to use family planning • Offer a reason to visit the clinic now, rather than procrastinate

Note: We made a systematic generation of hypotheses around the behavioural barriers to adolescent family planning decision-making and behaviour. We then collected qualitative evidence through clinic-based observations and qualitative interviews with married and unmarried adolescents, service providers, community mobilizers and local nongovernmental organization staff in three regions of Uganda in July 2018. We refined the hypotheses and prioritized them based on the evidence gathered to generate the insights and to inform the design objectives reported above.

## Methods

### Study design

We used a stratified, randomized controlled field trial to assess the intervention’s impact on the numbers of adolescents attending family planning clinics for services. We conducted the study in a network of 151 social franchise clinics which deliver sexual and reproductive health services in urban and peri-urban communities in Uganda (Box 1). Network clinics active for 6 months and meeting service quality standards were eligible for inclusion. We divided the resulting 126 clinics into 11 strata based on whether they accepted subsidized or free vouchers for services and on quartiles of mean adolescent family planning visits. We then randomized the clinics into intervention (66 clinics) and control (60 clinics) groups using a computer-generated list of random numbers. The intervention group was subsequently randomized into core (31 clinics) and core-plus (35 clinics) arms. Staff in all intervention clinics received core programme training and materials. Core-plus service providers additionally received training on provision of youth-friendly services. Control clinics did not receive intervention training or materials.

Box 1 **Setting for the study of a peer-referral family planning intervention, Uganda**

MSI Reproductive Choices is a nongovernmental organization providing sexual and reproductive health services in 37 countries. Marie Stopes Uganda provides more than half of contraception distributed nationally through multiple channels targeting underserved populations. Marie Stopes Uganda’s BlueStar network of 151 social franchise clinics delivers sexual and reproductive health services in urban and peri-urban communities. The organization provides training, equipment and support to franchised private-sector providers who are required to meet quality standards. Community-based mobilizers, supported by Marie Stopes Uganda, raise awareness about clinic services and generate referrals to services.

About two thirds of BlueStar clinics, generally in lower-income areas, participate in a programme funded by the United Kingdom Foreign, Commonwealth and Development Office. The programme provides vouchers which allow women to receive a short-term or long-acting reversible contraceptive of their choice. The clinics provide counselling, contraceptive insertion, side-effect management and contraceptive removal, and are reimbursed for service costs. Community health mobilizers distribute youth vouchers free to girls and young women under 25 years and sell paid discount vouchers to women of all ages for 2000 Ugandan shillings (about 0.55 United States dollars). The discount is a substantial amount from the full price of services typically not offered free unless supported by special programmes.

The sample size was constrained by the number of clinics, pre-intervention administrative data and the months in which implementation was funded. We estimated a minimum 10.8% detectable increase in number of visits by adolescent clients from baseline in the pooled intervention arm, with 80% power and an *α* of 0.05. The study protocol was approved by the independent ethics review committee of MSI Reproductive Choices and the TASO Uganda institutional review board (Pan African Clinical Trial Registry number: PACTR202012522031174).

### Intervention

The core intervention is primarily a peer-referral system that formalizes word-of-mouth means of advocating for family planning and is intended to reduce stigma about contraceptive use and normalize information-sharing among adolescents.^13^ More information about the theory of change behavioural mechanisms behind the intervention are available in the authors’ data repository.^14^ Girls aged 15–19 years who use contraceptives or have received counselling are given a refer-a-friend card by a family planning provider or community mobilizer, to give to a friend who is not currently using contraceptives. The friend redeems her card at network clinics for two friendship wristbands (one for her and one for the friend who referred her) and free contraceptive counselling. Girls are not required to accept counselling or family planning services to receive wristbands. Clinic service providers are instructed to provide wristbands at the start of the visits so that a girl presenting a refer-a-friend card does not feel pressured to stay for services. Refer-a-friend cards can be redeemed regardless of whether the girl has a voucher for additional services.

The programme offers girls an immediate motive to talk to friends about family planning, share advice and articulate reasons why girls like them may choose to use contraceptives. Girls who receive refer-a-friend cards get an endorsement from a trusted peer, empowering those who might otherwise feel uncomfortable about seeking services. When they offer the card to a friend, girls also have an opportunity to give advice that builds their confidence and solidifies their motivation to access family planning services when needed.^15^^,^^16^ When a girl visits a clinic to redeem a card and receives contraceptive counselling or services, she receives a new card to refer another friend, becoming an advice-giver herself. Materials in the facilities help to create an adolescent-friendly environment; these include posters welcoming adolescent girls and displays of redeemed refer-a-friend cards and badges worn by staff.

In addition to the intervention described above, service providers at a subset of facilities received a 3-day standard training in provision of youth-friendly services. This training builds knowledge, skills and capacity for service providers to deliver quality and appropriate sexual and reproductive health services to young clients in a way that respects their dignity, privacy and autonomy to make an informed choice. The training also builds service providers’ confidence and reaffirms a commitment to serve youth. More details of the intervention components have been published elsewhere.^13^

### Implementation

Materials for the intervention were distributed in January 2020 (Box 2). All intervention clinics launched the intervention by February 2020, the first full month of implementation. In April 2020, a decision was made to pause the programme due to restrictions on travel and public activity during the coronavirus disease 2019 (COVID-19) pandemic that began in late March 2020. However, the network clinics remained open to provide essential services. During the pause, girls could redeem refer-a-friend cards, but staff halted the card distribution. Once the intervention could be relaunched safely, we replenished materials and reminded staff about the programme protocols. Implementation restarted from August to October 2020.

Box 2 **Timeline of implementation of the peer-referral and clinic welcome family planning intervention for adolescents, Uganda, January to October 2020**

**January 2020 – Recruiting and training**

- Recruited and trained: trainers (1 week); community health mobilizers (1 week); service providers in core intervention group (1 week); and service providers in core-plus intervention group (1 week)
- Conducted concurrent training on youth-friendly services for core-plus intervention group (2 weeks)

**February to April 2020 – First implementation period**

Intervention delivery (3 months)

- Launched intervention in randomly selected eligible clinics and their surrounding areas
- In the community: community health mobilizers handed out refer-a-friend cards to satisfied contraceptive users they identified through their regular activities. Adolescent girls gave the cards to their friends who are not using family planning
- In the clinic: girls redeemed refer-a-friend cards in exchange for promotional items. While there, girls were exposed to intervention materials creating a youth-friendly environment. After counselling or uptake, girls received a refer-a-friend card of their own

Data collection

- Collected monthly outcome data
- Collected monthly data on refer-a-friend card distribution and redemption
- After first month, carried out interviews at the clinic with adolescent girls, service providers and community mobilizers. Carried out observations of facilities and community mobilizers

**May to July 2020 – Intervention paused**

Prepare for relaunch

- Conducted refresher trainings on-site (3 weeks)
- Ordered hand sanitizer, replenished intervention materials as needed (2 weeks)
- Intervention posters remained hanging on clinic walls, and clinics continued to redeem refer-a-friend cards with existing supplies, but card distribution was halted

Data collection

- Collected monthly outcome data

**August to October 2020 – Second implementation period**

Intervention delivery (3 months)

- Instituted new COVID-19 safety measures; otherwise, intervention activities were same as first period
- In the community: distribution of cards resumed
- In the clinic: redemption of refer-a-friend cards continued and distribution of cards resumed

Data collection

- Collected monthly outcome data
- Collected monthly data on refer-a-friend card distribution and redemption
- After second month post-relaunch, carried out interviews and observations, as before

COVID-19: coronavirus disease 2019.Note: Core facilities received the intervention package (adolescent peer-referral system and family planning clinic welcome materials); core-plus facilities received the intervention package and training on provision of youth-friendly services.

We collected routine service data from all facilities during a baseline period of 15 months before the intervention (November 2018 to January 2020) and through the 6-month implementation period (February to April and August to October 2020). Separately, intervention clinics and mobilizers manually submitted monthly reports of the refer-a-friend cards distributed and redeemed as part of the process evaluation.

Research assistants conducted observations and interviewed clients, service providers and mobilizers at a subset of facilities once per implementation period as part of a process evaluation to assess implementation fidelity and how the intervention was received and understood (more information on the process evaluation outcomes and indicators is available in the data repository).^14^

### Outcomes

The primary outcomes were continuous measures that clinics routinely report to Marie Stopes Uganda on a monthly basis: (i) number of visits for family planning services by adolescents aged 15–19 years and (ii) proportion of total family planning visits by adolescents. We selected these outcomes as the most reliable indicators available to assess the uptake of family planning services. Family planning services refers specifically to uptake of contraceptive methods, review of care or contraceptive removal. However, data from Marie Stopes Uganda indicated that the family planning services most commonly accessed by adolescents are contraceptive uptake, and only a small proportion of visits are for contraceptive removal. We also collected data on the monthly number of visits for family planning services by young adolescent girls aged 10–14 years and by young women aged 20–24 years as secondary outcomes to investigate potential spillover into untargeted age groups. 

### Analysis

We used the monthly visits by all family planning clients and by those aged 15–19 years to calculate the proportion of visits by adolescents per clinic for each month of data available. We made the descriptive and regression analyses using Stata version 14.2 (StataCorp, College Station, United States of America). We conducted analysis of covariance and *χ^2^* tests to compare primary outcomes and clinic characteristics, respectively, across the treatment arms at baseline. We then compared the mean and standard deviation (SD) number of visits during pre-intervention and intervention periods within each treatment arm using pairwise *t*-tests. We conducted multivariate linear regressions controlling for region, month, year, voucher status, randomization strata and unobserved facility fixed effects, to assess the impact of the intervention on primary outcomes.^17^ However, we found that a negative binomial regression model with the same controls was a superior fit for the high variation in counts of adolescent visits per clinic and therefore was the primary specification for that outcome reported here.^18^ We took the exponents of coefficients from that model to produce incidence rate ratios (IRRs) and 95% confidence intervals (CIs); the average treatment effect per clinic was estimated when controlling all other variables. All models included robust standard errors adjusted for clustering by facility. We excluded missing clinic months of data (1% of total) from the analysis. For the primary analysis, we estimated impact within the combined intervention group. Pre-specified secondary analyses explored differential impacts on primary outcomes by treatment arm, interaction effects between the intervention and voucher status, as well as average treatment effects on secondary outcomes.

Our main analysis excluded the 3 months during which implementation was suspended. However, for robustness we considered as intervention months: (i) the full period after launch, including suspension, because girls could still redeem pre-distributed refer-a-friend cards, and (ii) the first 2 months of implementation alone, before the countrywide lockdown and disruptions.

## Results

**Fig. 1:**
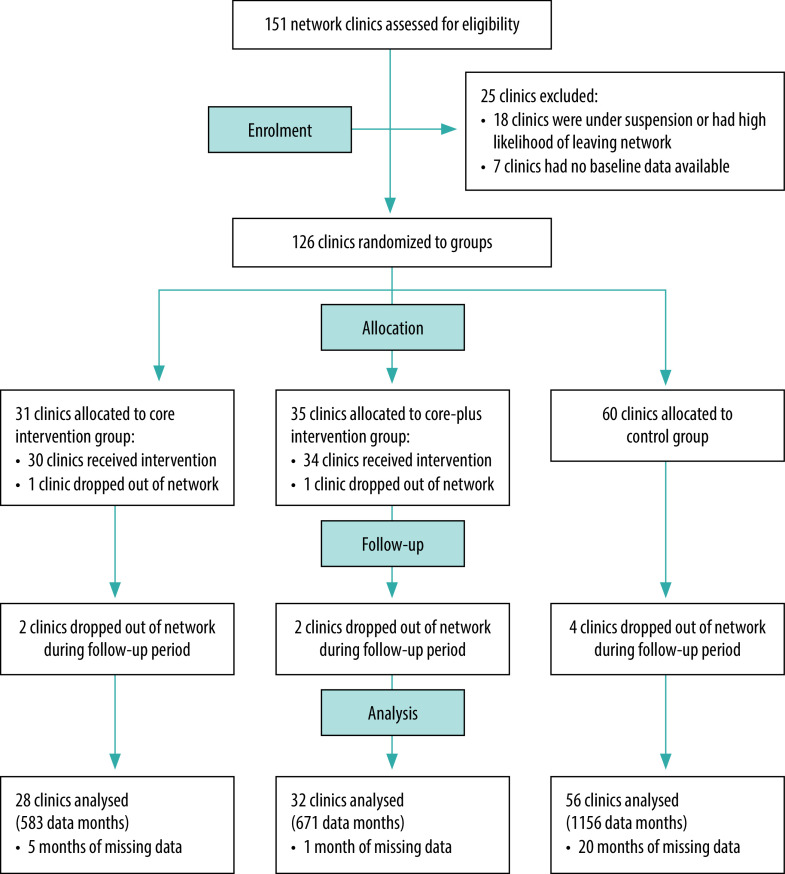
Flow diagram for the stratified, randomized controlled field trial of the peer-referral and clinic welcome family planning intervention for adolescents, Uganda, 2020

Ten clinics dropped out of the network between randomization of the 126 network clinics and the final analysis, leaving a final sample of 56 control clinics and 60 intervention clinics (28 core intervention clinics and 32 core-plus intervention clinics; Fig. 1). From the 24-month period November 2018 to October 2020 we obtained a mean of 23.8 months (SD: 0.4) of data per intervention clinic and 23.6 months (SD: 0.9) per control clinic (*P* = 0.06). Missing clinic months due to temporary pauses in clinic services appeared unrelated to which arm clinics were assigned to. Despite variation in primary outcomes between clinics and over time, the baseline characteristics of clinic groups were not significantly different by treatment arm (Table 2).

**Table 2:** Baseline characteristics of sexual and reproductive health clinics in the pre-intervention period, by treatment arm, Uganda, November 2018 to January 2020

Variable	Control clinics (*n* = 56)	Core intervention clinics (*n* = 28)	Core-plus intervention clinics (*n* = 32)	*P*
**Family planning client visits**				
Total no. of visits	104 225	47 044	56 059	NA
Mean (SD) monthly total no. of visits	126.3 (104.5)	112.3 (84.8)	116.8 (110.5)	0.19
Total no. of visits by adolescents	17 735	7 567	7 850	NA
Mean (SD) monthly no. of visits by adolescents	21.5 (27.0)	18.1 (20.8)	16.4 (23.0)	0.09
Mean monthly proportion of visits by adolescents, %	15.5 (12.1)	15.8 (12.6)	11.9 (10.4)	0.66
**Type of voucher system used,^a^ no. (%) of clinics**				0.48
No vouchers	21 (37.5)	7 (25.0)	13 (40.6)	
Paid vouchers	14 (25.0)	12 (42.9)	9 (28.1)	
Youth vouchers	21 (37.5)	9 (32.1)	10 (31.3)	
**Region, no. (%) of clinics**				0.80
Central	17 (30.4)	8 (28.6)	10 (31.3)	
Eastern	8 (14.3)	7 (25.0)	3 (9.4)	
Northern	9 (16.1)	4 (14.3)	5 (15.6)	
Western	22 (39.3)	9 (32.1)	14 (43.8)	

NA: not applicable; SD: standard deviation.

^a^ Vouchers allowed women to receive a short-term or long-acting reversible contraceptive of their choice at participating clinics. Community mobilizers distribute youth vouchers free to girls and young women aged under 25 years and sell paid, discounted vouchers to women of all ages.

Note: *P* values reflect analysis of covariance for primary outcomes and χ^2^ tests for voucher status and region. *n* is the number of clinics per treatment group. Core facilities received the intervention package (adolescent peer-referral system and family planning clinic welcome materials); core-plus facilities received the intervention package and training on provision of youth-friendly services. Pre-intervention period was November 2018 to January 2020.

### Uptake of family planning

Women of any age made a total of 207 328 family planning visits to the 116 study clinics in the 15-month pre-intervention period and 63 184 visits in the 6-month intervention period; 33 152 of the pre-intervention visits and 15 802 of the post-intervention visits were by adolescents. The monthly averages showed a consistent decrease in the number of family planning visits from a mean of 120.3 (SD: 102.0) before the intervention to 92.1 (SD: 80.2) after the intervention. Adolescents were the exception to that trend. The mean monthly proportion of visits by adolescents increased from 13.7% to 22.8% (*P* < 0.001) in intervention clinics and from 15.5% to 19.2% (*P* < 0.001) in control clinics before and after the intervention. However, only the intervention clinics recorded a significant increase in the mean monthly number of adolescent family planning visits, from 17.2 (SD: 22.0) to 23.4 (SD: 31.2; *P* < 0.001) before and after the intervention. In control clinics the mean monthly numbers of visits by adolescents were 21.5 (SD: 27.0) and 22.7 (SD: 34.5) before and after the intervention, respectively (*P* = 0.54; Table 3).

**Table 3:** Pairwise comparison of client visits to sexual and reproductive health clinics in pre-intervention (15 months) versus intervention (6 months) periods, by treatment arm, Uganda, 2018–2020

Group	Total no. of family planning visits	Mean (SD) monthly no. of visits	Total no. of visits by adolescents	Mean (SD) monthly no. of visits by adolescents	Mean (SD) proportion of visits by adolescents, %
**Control clinics (*n* = 56)**					
Pre-intervention period	104 225	126.3 (104.5)	17 735	21.5 (27.0)	15.5 (12.1)
Intervention period	30 706	92.8 (78.9)	7 499	22.7 (34.5)	19.2 (15.2)
***P*** value	NA	< 0.001	NA	0.54	< 0.001
**All intervention clinics (*n* = 60)**					
Pre-intervention period	103 103	114.7 (99.3)	15 417	17.2 (22.0)	13.7 (11.6)
Intervention period	32 478	91.5 (81.5)	8 303	23.4 (31.2)	22.8 (17.2)
*P* value	NA	< 0.001	NA	< 0.001	< 0.001
**Core intervention clinics (*n* = 28)**					
Pre-intervention period	47 044	112.3 (84.8)	7 567	18.1 (20.8)	15.8 (12.6)
Intervention period	13 639	83.2 (64.1)	3 533	21.5 (27.5)	24.9 (18.3)
*P* value	NA	< 0.001	NA	0.09	< 0.001
**Core-plus intervention clinics (*n* = 32)**					
Pre-intervention period	56 059	116.8 (110.5)	7 850	16.4 (23.0)	11.9 (10.4)
Intervention period	18 839	98.6 (93.5)	4 770	25.0 (34.1)	21.1 (15.9)
*P* value	NA	< 0.05	NA	< 0.001	< 0.001

NA: not applicable; SD: standard deviation.

Notes: *P* values are based on pairwise *t*-tests. *n* is the number of clinics per treatment group. Core facilities received the intervention package (adolescent peer-referral system and family planning clinic welcome materials); core-plus facilities received the intervention package and training on provision of youth-friendly services. Pre-intervention period was November 2018 to January 2020. Intervention period was February to April and August to October 2020.

In adjusted regression models we estimated average effects when the core and core-plus interventions were combined or separated. In our primary analysis, we found statistically significant intervention effects on both primary outcomes relative to the control group when combined (Table 4). The negative binomial regression (model 1) showed a significant nonlinear effect on the monthly number of visits by adolescents (IRR: 1.45; 95% CI: 1.14–1.85), corresponding to an estimated 45% increase in visits. Controlling for all other variables, this percentage translates to over five additional adolescent client visits per clinic per month on average. We also found that the number of adolescents as a proportion of the total number of family planning visits (model 3) increased significantly in intervention clinics by 5.3 percentage points (*β*: 0.053; 95% CI: 0.020–0.087). When looking at the different impact by treatment arm (model 2), we found that only the core-plus intervention significantly predicted numbers of adolescent visits (IRR: 1.62; 95% CI:1.21–2.17). The estimated 62% increase in visits translates to over seven additional adolescents per month in participating clinics. The core intervention alone could be linked to a significant increase in the adolescent proportion of visits, but its effect on the number of visits by adolescents did not reach significance (IRR: 1.26; 95% CI: 0.97–1.65). This 26% increase translates to around three additional adolescents attending clinics per month. 

**Table 4:** Estimated treatment effects of the peer-referral intervention on primary outcomes relative to control, Uganda, 2020

Variable	No. of family planning visits by adolescents			Proportion of family planning visits by adolescents	
	Negative binomial regression			Linear regression	
	Model 1	Model 2		Model 3	Model 4
	IRR (95% CI)	IRR (95% CI)		β (95% CI)	β (95% CI)
Average intervention effect	1.45 (1.14–1.85)	NA		0.05 (0.02–0.09)	NA
Core intervention effect	NA	1.26 (0.97– 1.65)		NA	0.05 (0.00–0.10)
Core-plus intervention effect	NA	1.62 (1.21– 2.17)		NA	0.05 (0.02–0.09)
No. of clinic months analysed	2410	2410		2410	2410

CI: confidence interval; IRR: incidence rate ratio (exponentiated regression coefficients); NA: not applicable.

Notes: We estimated regression models with robust standard errors and adjusted for clustering by facility. All models controlled for type of clinic vouchers used (youth vouchers, paid vouchers or no vouchers), region, strata used for randomization, and time fixed effects (see the authors’ data repository).^14^ Core facilities received the intervention package (adolescent peer-referral system and family planning clinic welcome materials); core-plus facilities received the intervention package and training on provision of youth-friendly services.

### Referral card redemption

Mobilizers and service providers from each intervention clinic were expected to submit monthly reports on the distribution and redemption of refer-a-friend cards to Marie Stopes Uganda. However, about 40% (292 reports) of the 720 expected reports were not submitted; only 44 clinics submitted at least two complete (including both the provider and mobilizer component) manual reports during the implementation months. We believe this is a reporting issue, rather than an indicator of low compliance with the intervention. We noted that providers and mobilizers continued to request additional cards to distribute, and clinic staff making routine site visits observed that numerous redeemed cards were displayed in clinic counselling rooms. Thus, the recorded numbers of refer-a-friend cards distributed (12 826 cards) and redeemed (5477 cards) are underestimates of the reach of the intervention. 

### Secondary analyses

Results for the secondary analyses are presented in the data repository.^14^ Although refer-a-friend card distribution was paused during the 3-month break in the study, the effects noted above remained statistically significant when including those months in the intervention period. The effects of the intervention remained statistically significant and were greater when restricting the intervention period to the 2 months before the COVID-19 lockdown. While the availability of youth vouchers significantly predicted the numbers of visits by adolescents, a positive although non-significant interaction suggested that the intervention may have greater impact where subsidized service vouchers are available. We did not find significant effects of the intervention on the average monthly number of visits by younger (age 10–14 years) or older (age 20–24 years) youth clients.

## Discussion

Despite interruption due to the COVID-19 pandemic, we found a significant effect of this peer-referral intervention on both of the primary outcomes: average monthly number of visits by adolescents and proportion of total visits by adolescents. The magnitude of the effects was reduced when we included data from the months when the programme was paused; nevertheless, the data still showed a significant impact of the intervention, providing some evidence for the potential resilience and endurance of the intervention. A review of previous studies evaluating the reach of adolescent interventions in low-income settings suggests that many adolescents are not being reached by the sexual and reproductive health programmes intended for them.^10^ We received positive feedback during the process evaluation^19^ from enthusiastic service providers, community mobilizers and clients, who wanted the intervention to continue. Our experience therefore supports the success of the programme at connecting hard-to-reach adolescent girls to valuable family planning counselling and services, and demonstrates the intervention’s acceptability, appropriateness and ease of implementation.

Negative stereotypes and social stigma around contraception and sexual relationships can inhibit uptake of family planning.^20^ Interventions aimed at adolescents may therefore be especially important. The refer-a-friend card intervention aims to facilitate advice and experience-sharing among peers, and to make visiting clinics for accurate guidance on family planning options less intimidating. The referral cards, staff nametags and clinic posters aim to make girls feel invited and welcome at facilities, a sentiment expressed by girls during process evaluation interviews.

The inclusion of youth-friendly services training in the core-plus treatment arm appeared to show the strongest impact, increasing the monthly number of adolescent visits by 62% compared with the control. Although our study did not evaluate the impact of the training alone, there is little evidence in the literature that the training itself increases the uptake of adolescent family planning.^21^^–^^24^ Thus our findings may suggest the importance of youth-friendly service provision when paired with interventions to facilitate adolescent demand for and access to clinic services. It should be noted that youth-friendly services training was previously conducted in 2017 for network clinics entering the youth voucher programme (about one third of clinics), so it is possible that a subset of service providers in all study arms had earlier exposure to the training. 

Clinics with youth vouchers allowing access to free family planning methods had greater baseline numbers of adolescent clients. Such clinics may be better prepared for peer referral, with staff more experienced in provision of youth services. Although not a statistically significant effect, the intervention’s positive interaction with vouchers suggests that efforts meant to close gaps between intentions and actions and generate demand for services would be best paired with measures to increase affordability. Indeed, service providers in clinics without youth vouchers reported that many girls coming in for counselling could not afford to pay for their desired family planning methods. However, our results suggest a significant impact of the intervention on uptake even when services cannot be offered for free.

The pause in the intervention due to the COVID-19 pandemic is an obvious limitation of the study. However, the randomized controlled study design and our robustness checks helped to mitigate the disruption. The pandemic may have affected demand for and delivery of family planning services, and hence the effectiveness of the intervention, both positively and negatively. However, we found no evidence that the effects differed between clinics in intervention and control groups. Schools remained closed in Uganda from March 2020 throughout the study period. Free time and restricted movement due to transport shutdowns or close parental supervision may have affected both adolescents’ need for and ability to access family planning services. Furthermore, both the Ugandan health ministry and Marie Stopes Uganda increased their outreach services to adolescents during the study period, pairing family planning messaging with COVID-19 posters and radio broadcasts with advice to the public. Such escalated efforts across the country may have attenuated the intervention’s effect. Social distancing restrictions may also have led to limited opportunities for referral of a friend. Stronger effects observed during the early months of implementation suggest the pandemic may have weakened average intervention effects. Finally, although introducing new data reporting methods is difficult under normal circumstances, operational challenges during the pandemic context may have further contributed to low reporting rates of the refer-a-friend cards distributed and redeemed by intervention clinics. These low reporting rates are an unfortunate limitation to assessing the full extent of participation in the programme beyond the routine service data collected. When possible, reporting procedures should be adapted to the reality of clinic work, to facilitate monitoring of adherence to the intervention.

Another limitation is that routine service reporting did not record how many adolescents received family planning counselling without uptake of a contraceptive method; the primary outcomes therefore reflect family planning uptake only. Average intervention effects of over five new adolescent family planning clients per clinic per month suggests that nearly 2000 additional girls were served across six implementation months. Yet the process evaluation revealed that many girls redeemed refer-a-friend cards for wristbands and counselling without taking up contraception. Underreported card redemptions suggest that at least twice as many adolescents may have benefited from counselling alone. Considering that many adolescents who are not yet sexually active are likely to become sexually active soon, early counselling may be a gateway to accessing family planning services once needed. Girls remarked on the value of the counselling to them and their friends, and expressed their intention to eventually return for services. No evidence from the process evaluation suggests girls felt pressured into taking up family planning when redeeming their refer-a-friend card for wristbands. The greater number of cards redeemed compared with the number of clients served further suggests the intervention did not undermine the girls’ choices and actions. Unfortunately, we were unable to study whether the intervention influenced the distribution of contraceptive methods taken up by adolescents because the routine service data did not disaggregate this variable by age group.

In conclusion, this trial provides evidence that a behavioural change intervention designed to target identified adolescent barriers can increase their uptake of family planning counselling and services, even during a global pandemic. While behavioural interventions tailored to a specific context are not always generalizable, the behavioural design approach itself is a generalizable and robust process to intervention development. The behavioural diagnosis underlying this intervention identified barriers to adolescent uptake that may be relevant beyond the study network and beyond Uganda, suggesting these designs might be adapted to similar settings where family planning services are available but similar barriers inhibit girls’ access.
